# Risk evaluations and condom use decisions of homeless youth: a multi-level qualitative investigation

**DOI:** 10.1186/s12889-015-1419-9

**Published:** 2015-01-31

**Authors:** David P Kennedy, Ryan A Brown, Penelope Morrison, Loryana Vie, Gery W Ryan, Joan S Tucker

**Affiliations:** RAND Health, RAND Corporation, P.O. Box 2138, 1776 Main Street, Santa Monica, CA 90407 USA; The RAND-University of Pittsburgh Health Institute, Pittsburgh, PA USA; The Department of Psychology, University of Pennsylvania, Philadelphia, PA USA; The Department of Psychology, University of California, Riverside, CA USA

**Keywords:** Condoms, Homeless youth, Heterosexual sex, HIV, Reproductive health, Qualitative methods

## Abstract

**Background:**

Homeless youth are at higher risk for sexually transmitted infections and unwanted pregnancy than non-homeless youth. However, little is known about how they evaluate risk within the context of their sexual relationships. It is important to understand homeless youths' condom use decisions in light of their sexual relationships because condom use decisions are influenced by relationship dynamics in addition to individual attitudes and event circumstances. It is also important to understand how relationship level factors, sexual event circumstances, and individual characteristics compare and intersect.

**Methods:**

To explore these issues, we conducted semi-structured interviews with 37 homeless youth in Los Angeles County in 2011 concerning their recent sexual relationships and analyzed the data using systematic methods of team-based qualitative data analysis.

**Results:**

We identified themes of risk-related evaluations and decisions at the relationship/partner, event, and individual level. We also identified three different risk profiles that emerged from analyzing how different levels of risk intersected across individual respondents. The three profiles included 1) Risk Takers, who consistently engage in risk and have low concern about consequences of risk behavior, 2) Risk Avoiders, who consistently show high concern about protection and consistently avoid risk, and 3) Risk Reactors, those who are inconsistent in their concerns about risk and protection and mainly take risks in reaction to relationship and event circumstances.

**Conclusions:**

Interventions targeting homeless youth should reflect multiple levels of risk behavior and evaluation in order to address the diversity of risk profiles. Relationship/partner-, event-, and individual-level factors are all important but have different levels of importance for different homeless youth. Interventions should be tailored to address the most important factor contributing to homeless youth reproductive needs.

**Electronic supplementary material:**

The online version of this article (doi:10.1186/s12889-015-1419-9) contains supplementary material, which is available to authorized users.

## Background

Homeless youth between the ages of 13–24 continue to be a highly vulnerable population for a variety of sexual health risks. Homeless youth are far more likely than housed youth to be infected with the human immunodeficiency virus (HIV) [1,2] and other sexually transmitted infections (STIs) [3]. The prevalence of pregnancy among homeless girls is estimated at 35-45%, which is much higher than the national average [3-6]. Greater use of the male condom would help prevent many of the sexual and reproductive health risks faced by homeless youth [7-9]. However, rates of condom use are much lower than necessary to prevent the spread of infection and lower rates of unwanted pregnancies among homeless youth. Studies have found that 40-70% of homeless youth report engaging in unprotected sexual intercourse [10-13].

The purpose of this paper is to generate empirical evidence to inform the development of reproductive health interventions to increase condom use among homeless youth. We aim to inform interventions that target reductions in both STIs and unwanted pregnancies; therefore we focus on homeless youth who engage in heterosexual sex. The theoretical framework guiding our data collection and analysis is the social ecological model of development in which human behavior is the result of individual characteristics as well as multiple, nested environmental contextual forces [14]. As a guiding theoretical framework for informing intervention development, an ecological approach acknowledges that: 1) understanding and intervening in health behavior requires a focus on multiple environments (e.g., social, cultural, organizational, physical) in which individual behaviors are nested; and 2) interventions should focus on specific behaviors and address the multiple levels of influence on these behaviors [15]. Our theoretical approach also recognizes that understanding and intervening on condom use requires an emphasis on the dyadic context of sexual behavior because condom use inherently involves the behaviors and interactions between two people (i.e. are dyadic and relational) [16]. Beyond merely recognizing the relationship context of particular unprotected sex events, our approach recognizes that individuals have multiple relationships and, therefore, dyadic influences on unprotected sex are nested within individual-level factors. Also, individual unprotected sex events are themselves nested within the dyadic context. This is because the characteristics of relationships change over time, and factors such as evaluation of risk and dynamics between partners change over time, which impact decisions about protection during specific sexual events. In addition, attention to factors associated with specific events recognizes that circumstances of sexual events can at times outweigh individual or dyadic level factors.

**Figure 1:**
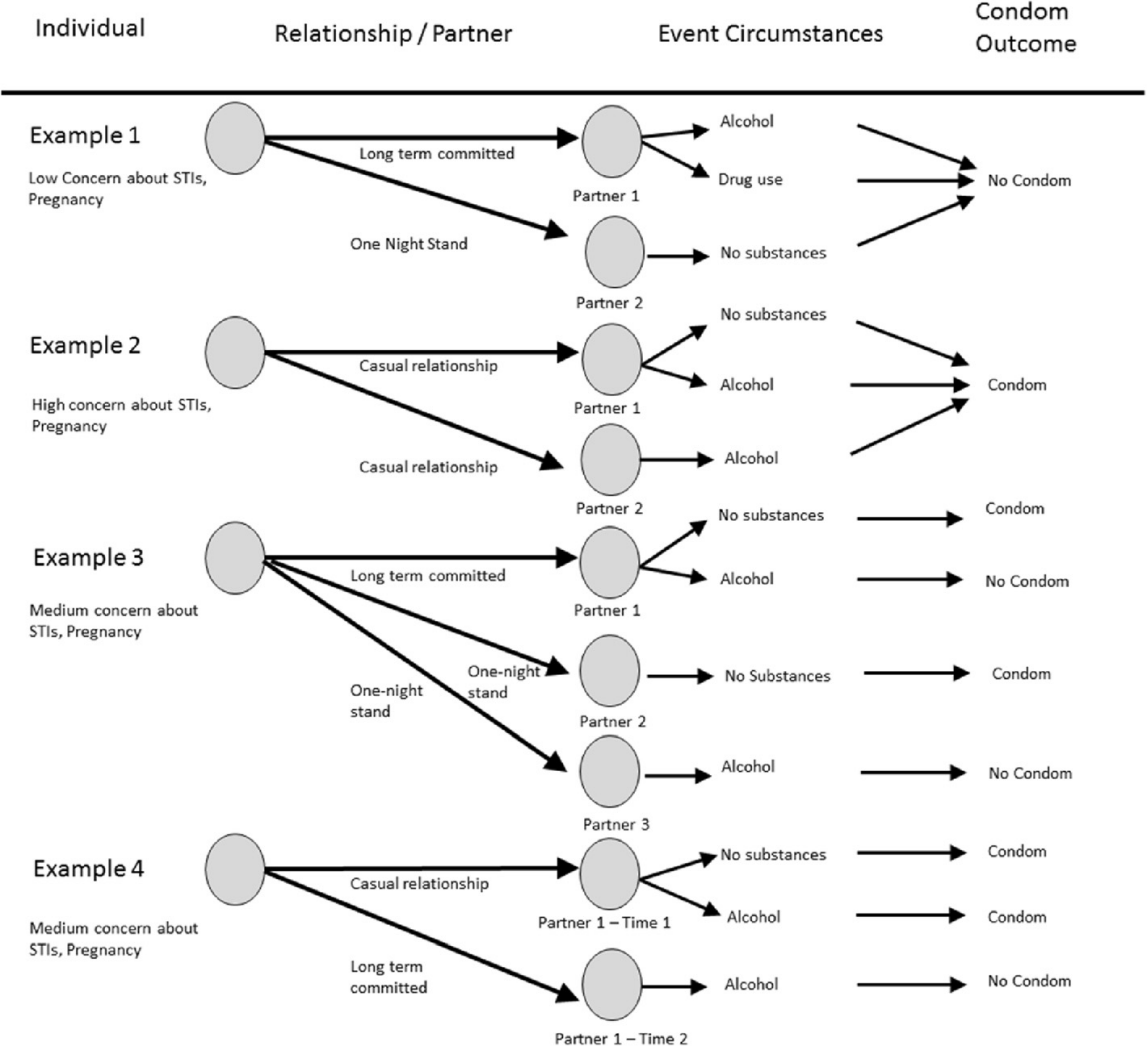
**Four examples of multi-level factors involved in condom use decisions and risk evaluation.** The examples illustrate how different factors influence condom use in different ways. The figure depicts four examples of combinations of individual, relationship/partner, and event circumstances that provide hypothetical context of condom use or non-use. Circles depict example individuals and their partners. Example individuals are labeled with the amount of concern they have for STI risk or unwanted pregnancy (high, medium, low). Arrows between example individuals and their partners are labeled for the type of relationship (Long-term committed, casual, one-night stand). For each partner, arrows depict multiple sex events with the partners with different substance use during the events (alcohol, drug, no substance use). An arrow for each event indicates if condoms were used during the event.

Figure 1 illustrates how these nested factors interact in four hypothetical examples. The four examples depict hypothetical combinations of individual, relationship/partner, and event circumstance levels of influence on condom use or non-use. The first example depicts an individual who has low concern about STIs/pregnancy and does not use condoms regardless of partner type or event circumstance. In contrast, the second example depicts an individual with a high level of concern about STIs/pregnancy who always uses condoms regardless of partner type and event circumstances. The third and fourth examples depict individuals with moderate concern about STIs/pregnancy and use condoms depending on relationship and event circumstances. The third individual engages in condom use with different types of partners except when drinking alcohol. The fourth example depicts an individual with one partner at two stages of their relationship, where condom use ceases as the relationship transitions from casual to committed. Together, these hypothetical cases depict the complicated intersections of nested factors that may intersect in different ways to influence condom use decisions. Developing interventions to help homeless youth lower their risk of STIs and unwanted pregnancies requires an understanding of how their lives trigger different risk factors that lead to different pathways of risk and protective behaviors.

### Existing condom use interventions for homeless youth

According to a recent review, existing condom use interventions for homeless youth are limited [17]. This review identified only three homeless youth intervention studies with condom use outcomes that were rigorously evaluated [1,18,19]. These interventions were guided by ecological principles and target multiple levels of influence on condom use, including individual-level characteristics (e.g. increasing knowledge about consequences of unprotected sex, building skills to assess risk and engage in safe sex), structural factors (e.g. increasing the capacity of homeless youth services to address reproductive health needs of homeless youth, such as making condoms available, and providing training about reproductive health to counselors), and social factors (engaging families and extended social networks/communities of homeless youth in interventions). The review concluded that these studies had significant limitations (e.g. selection and attrition bias, lack of power) and that there is a need for novel intervention development. Another limitation of these interventions is that none of them explicitly address the dyadic level of influence on unprotected sex. This is not unusual as most programs to promote safer sex in the United States intervene exclusively on factors within the individual [16,20]. Research findings demonstrating the importance of the relationship context of unprotected sex (e.g. [21]) has led to arguments that intervention efforts could be improved by incorporating a more explicit focus on the dyadic and relationship level influences on unprotected sex [22,23]

### Unprotected sex in heterosexual homeless relationships

Although investigations of the sexual and romantic relationships of homeless populations have been limited [24], recent studies of unprotected sex among heterosexual homeless adults and youth confirm the importance of the dyadic context of homeless unprotected sex. Several recent analyses of predictors of unprotected sex with particular partners have demonstrated that partner and relationship characteristics are associated with unprotected sex controlling for other factors, such as individual attitudes about condoms, concerns about pregnancy and STIs, drug and alcohol use, and social network characteristics [25-27]. Recent qualitative studies of specific sexual events of homeless men, women and youth also demonstrate the importance of the romantic relationship context of decision making about protection during sexual events [28-30]. In these studies, homeless men, women and youth described unprotected sex events involving partners with whom they had used condoms in the past but switched to unprotected sex after their relationship developed a strong emotional bond.

Many studies of reproductive health factors relevant for heterosexual couples have emphasized the impact of gendered power differentials on condom negotiation [31], as well as the association between communication about protection prior to sex and condom use [20]. However, these recent studies of unprotected sex in homeless populations have not supported the assumptions that imbalances of power are primary drivers of unprotected sex in these relationships and that greater communication will lead to greater condom use. Although examples of sexual coercion and deference to male partner preferences for unprotected sex were noted in these studies, homeless women and girls also described how feelings of trust and safety with their male partners often led them to initiate unprotected sex. In addition, homeless men and boys at times described their vulnerability and powerlessness in their sexual relationships with women [29,32]. One study also found quantitative and qualitative evidence that communication about risk prior to sex was associated with unprotected sex among homeless youth [25]. The results on the whole challenge assumptions based on studies of non-homeless heterosexual populations and present a complicated picture of the factors that trigger unprotected sex within heterosexual homeless relationships.

Although these studies provide insight into factors associated with unprotected sex among homeless populations, they have important limitations. First, while the quantitative findings identify associations between relationship characteristics and high risk sexual behavior, measures of condom use within relationships were based on overall ratings of frequency of condom use with the partner. However, the qualitative studies suggest that sexual relationships often do not have only one pattern of condom use. Within relationships, patterns of condom use change over time as the relationship evolves and feelings of relationship commitment increase. Also, because the design of these qualitative studies focused on specific recent sexual events, exploration of how the relationship trajectory prior to the sexual event influenced decisions about protection was limited. Another limitation to applying previous findings to heterosexual homeless couples is that these studies primarily focus on the disease risks of unprotected sex. However, qualitative studies of homeless respondents in heterosexual relationships present examples of respondents who discussed the key role that pregnancy concerns/desires and contraceptive use play in their decisions to begin engaging in unprotected sex with their partners [28,29,33].

### This study

In order to build on these findings and to better understand how factors at the relationship/partner-, event-, and individual-level influence condom use patterns of heterosexual homeless youth, we conducted exploratory semi-structured interviews with homeless youth in Los Angeles County. Our goal was to better understand the complex mechanisms influencing unprotected sex to inform the development of sexual and reproductive health interventions targeting high rates of STIs and unwanted pregnancy among homeless youth. We take an exploratory approach because developing an effective intervention strategy for a complex health outcome depends upon a detailed, nuanced, and accurate understanding of the multiple causal pathways involved in such behaviors [15]. Many intervention approaches to reducing sexual risk behavior among homeless populations focus narrowly on a limited set of causal factors, such as psychological elements of condom knowledge or motivation and skills for condom use [34,35]. This narrow approach to complex and interrelated problems that are deeply situated within relationships and social context may explain the limited success of health interventions with homeless populations [36,37]. Our aim in this study is to build theory from empirical data about the process of unprotected sex among homeless youth rather than investigate any one particular theoretical causal pathway.

## Methods

### Sampling and participants

We recruited homeless youth from one shelter, two drop-in centers, and four street sites in two areas of Los Angeles County with relatively large populations of homeless youth – Hollywood and Santa Monica/Venice Beach. The goal was to obtain roughly equal numbers of interviews from service sites vs. street sites, and a similar number of male and female respondents. We counted youth at each site and used a random number table to select youth for eligibility screening. Across all seven sites, 129 youth were counted and a total of 62 youth were sampled for eligibility screening.

Youth were eligible if they: a) were between the ages of 13–24; b) were not currently living with a parent or guardian; c) were not getting most of their support for food and housing from family or a guardian; d) spent the previous night in a shelter, outdoor or public place, hotel or motel room rented with friends (because of no place else to go), or other place not intended as a domicile; e) were English speaking; and f) engaged in vaginal or anal sex during the past 6 months with at least one opposite sex partner. A total of 20 youth out of the 62 screened were found to be ineligible due to not fitting our formal definitions of homelessness (n = 3), falling outside the 13–24 year age bracket (n = 4), and not having had heterosexual sex in the last three months (n = 13). Additionally, one youth was found to be a repeat respondent. Of the remaining 41 eligible youth, two refused the interview and two failed to complete the protocol, leaving a total of 37 completed interviews - 17 males and 20 females (66% eligibility rate, 90% response rate). Youth were paid $25 for their participation. The research protocol was approved by the Human Subjects Protection Committee (HSPC) of RAND and a Certificate of Confidentiality was obtained from the National Institutes of Health. The RAND HSPC waived the requirement of parental consent because the research involved no more than minimal risk to the subjects and could not practicably be carried out without the waiver.

### Interview protocol

Interviews consisted of a matrix-based protocol involving open ended questions and probes. First, interviewers asked respondents to list the sexual relationships they had with members of the opposite sex over the past 6 months and to indicate if these relationships involved 1 or more sexual encounters. Next, interviewers used the following decision rules to select 2 of these sexual relationships from the past 6 months with the goal of maximizing intra-case diversity in relationship type: 1) If respondents had at least 1 relationship with a single sexual encounter and 1 relationship with multiple encounters, the most recent of these two types was selected. 2) If recent relationships did not differ in this characteristic, the 2 most recent relationships were selected. 3) If respondents only had 1 sexual relationship in the past 6 months they were queried about only this relationship.

The semi-structured interviews were designed to explore respondents’ sexual relationship histories with their partners. For each partner, interviewers explored 6 main topic areas for each stage of the relationship. Relationship stages included: 1) general relationship history of the respondent, 2) first sexual experience with the partner, 3) the most recent sexual event, and 4) the first event that involved some transition in their protective behavior with the partner (e.g., the couple’s first unprotected sex experience after previous consistent condom use). The topics explored within each stage included: 1) context (e.g. setting of a sex event), 2) relationship/feelings, 3) alcohol and drug use (asked about separately), 4) evaluation of STI risk, 5) evaluation of pregnancy risk, and 6) evaluation of the relative importance of STI vs. pregnancy risk. We developed a grid-based matrix protocol to assist interviewers in conducting the semi-structured interviews. This technique maximizes interviewer flexibility within interviews as well as consistency across interviews and has been used in previous exploratory studies of the sexual experiences of homeless men [38] and women [28]. Two male and two female interviewers with experience interacting with homeless populations on sensitive topics were trained in general qualitative interview techniques as well as specific techniques for using the interview matrix. Interviewers and respondents were matched based on gender. All interview content was audio recorded and transcribed. Interviewers also wrote notes during interviews and developed more expanded field notes of the interview content immediately after each interview using Livescribe “smart” audio recording pens (www.livescribe.com). Both interview transcriptions and field notes provided the primary data for formal qualitative analysis.

### Qualitative data coding

We conducted a thematic analysis using Atlas.ti software and developed a codebook of themes using standard procedures for team-based qualitative analysis [39,40]. We started with three master *a priori* codes to tag instances of text related to levels of analysis that have been found to be associated with unprotected sex among homeless: (1) *Relationship/Partner-level influences* – emotional or behavioral patterns with a particular relationship or observations about a partner that influence risk, (2) *Event-level influences*– circumstances of particular sexual events related to decisions regarding risk within particular relationships, and (3) *Individual-level influences –* personal preferences of the respondent that influence risk decisions across their relationships. We identified sub-themes inductively by examining patterns of themes across respondents. We also compared behaviors and risk decision-making of particular respondents both within and across their relationships to identify patterns of risk evaluation. The lead author wrote a formal definition of the characteristics that appeared to distinguish groups of respondents’ patterns of risk evaluation. Two independent coders applied these definitions to the data for each case and classified respondents according to their risk characteristics. We calculated the Kappa statistic to measure agreement between coders in their classification of respondents, evaluated codes in cases where Kappa was lower than 0.70 [41], and revised coding definitions and re-classified respondents based on the revised code definitions. We discussed each discrepant code until we reached consensus on a complete set of codes including classifying each respondent into one of the three risk profiles.

## Results

See Table 1 for characteristics of the sample of 37 homeless youth we interviewed. Our initial coding confirmed the existence of three major types of influences on unprotected sex: 1) **relationship/partner-level influences** and two non-relationship influences including 2) (within relationship) **event-level influences** and 3) (across relationship) **individual-level influences**. Subsequent open coding helped identify several sub-themes within each major theme. Relationship/partner-level influences included 6 risk sub-themes: *emotional factors, relationship type, perceived partner preferences, communication about risk, perceived partner characteristic*s, and *relationship trajectory and risk.* Event-level influences included 2 sub-themes: *drug and alcohol use,* and *sexual arousal*. Individual-level influences included *concern about pregnancy and STIs* and *general attitudes about risk and typical patterns of risk engagement and protection.* Table 2 lists the three major themes, the 10 sub-themes, and descriptions of each sub theme. Our within-respondent analysis identified three overall risk/protection profiles: 1) Risk Takers: *high risk, low concern*, 2) Risk Avoiders: *low risk, high concern*, and 3) Risk Reactors: *medium risk, medium concern*. Table 3 provides details about these overall risk/protection profiles including details about how each sub-theme corresponded with the overall profile. Table 3 also provides Kappa values for each profile, indicating an acceptable level of coder agreement [41]. Below we describe these findings in detail. We also provide an extensive set of exemplary quotations illustrating each sub-theme in Table 4.

**Table 1:** **Sample characteristics**

	**Overall (n = 37)**		**Males (n = 17)**		**Females (n = 20)**	
	**Mean/count**	**SD**	**Mean/count**	**SD**	**Mean/count**	**SD**
Age in Years	19.8	2.5	20.4	2.2	19.3	2.7
Race						
Black	6		3		3	
White	17		8		9	
Native American/Alaskan Ntve	1		0		1	
Asian	1		0		1	
Multiracial	2		1		1	
Hispanic	10		5		5	
Education						
No HS/GED	18		10		8	
HS/GED	10		4		6	
Some college, no degree	8		2		6	
Technical certification	1		1		0	
Employment						
Unemployed	36		17		19	
Part time	1		0		1	
Age first left home (in years)	16.1	2.7	16.0	3.0	16.2	2.5
Years Homeless	3.7	2.3	4.4	2.6	3.1	1.8

**Table 2:** **Summary of themes resulting from analysis of individual, relationship/partner, and event circumstances levels**

**Relationship/Partner**		**Event circumstances**		**Individual**	
**Theme**	**Description**	**Theme**	**Description**	**Theme**	**Description**
*Emotional factors*	Comments about trust, familiarity with the partner, and developing an emotional connection with specific partners that influenced decisions to use condoms	*Drug and alcohol use*	Discussions of the influence of drugs and alcohol on the respondents experience and attitude towards unprotected sex	*Concern about Pregnancy and STIs*	Respondent’s concern or lack of concern about pregnancy and STI risk that influenced their risk taking decisions across different events and partners
*Relationship type*	Discussions of the relationship between categories or types of partners and risk evaluation/unprotected sex	*Sexual arousal*	Discussions of how momentary feelings of sexual arousal affected decision making about engaging in unprotected sex	*Concern about Pregnancy and STIs*	Respondent’s concern or lack of concern about pregnancy and STI risk that influenced their risk taking decisions across different events and partners
*Perceived partner preferences*	Decisions about protection that were dictated by the preferences of partners				
*Communication about Risk*	Descriptions of interactions between partners that lead to decisions to engage in or not engage in unprotected sex				
*Perceived partner characteristics*	Descriptions of calculations about risk based on perceived or observable characteristics of the partners that are related to increased or decreased risk of transmitting an STD				
*Relationship trajectory and risk*	Discussions about how the respondent and a particular partner either changed or did not change their pattern of protection over time and what affected the change in protection or what would or would not affect the pattern of their behavior with the partner

**Table 3:** **Summary of theme pattern among respondents in three risk/protection profiles identified in within-case analyses**

**Risk profile**	**Relationship/Partner**	**Event circumstances**	**Individual**
**Risk takers: High risk, low concern**	• *Emotional Factors*: No observed pattern. *Relationship Type*: No observed pattern.	• *Drug and Alcohol Use*: Frequent risky sex after drug use or for pleasure seeking but no observed pattern of risk-taking because of substance use. Low frequency in use of condoms if used at all.	• *Concern about Pregnancy and STIs:* Low concern about risks of pregnancy or disease. Often know they are at risk but not concerned even when having a history of pregnancy or disease. Commonly express fatalistic views of inability to prevent pregnancy or disease as a justification for not using protection.
	• *Perceived Partner Preferences*: Occasional examples of deference to partners who preferred to use protection (e.g. condoms, withdrawal).		
N = 12 (7 female, 5 male)	• *Communication about Risk*: No observed pattern.	• *Sexual Arousal:* No observed pattern.	• *General Attitudes about Risk and Typical Patterns of Risk Engagement and Protection*: Have a consistent pattern of risk taking and lack of concern about risk across different types of partners and event circumstances. Only occasional descriptions of protected events.
Kappa = .82	• *Perceived Partner Characteristics:* Rare discussions of concern about risk and use of protection with certain types of partners perceived to be more risky than the respondent. Dominant pattern of engaging in unprotected sex with all partners regardless of partner characteristics or history of STIs.		
	• *Relationship Trajectory and Risk:* No observed pattern.		
**Risk avoiders: Low risk, high concern**	• *Emotional Factors*: No observed pattern.	• *Drug and Alcohol Use*: Occasional descriptions of sexual events without use of protection explained by alcohol or drug use. These events are consistently described as exceptions and rare.	• *Concern about Pregnancy and STIs:* Often very concerned about both pregnancy and STI risk. Often discuss specific consequences of risk behavior, such as having a child when not ready or pain or stigma caused by STIs. Often use multiple strategies to avoid risk including using condoms in addition to other forms of birth control. Express regret for the times they did not use condoms.
	• *Relationship Type*: No observed pattern.		
N = 10 (3 female, 7 male)	• *Perceived Partner Preferences*: No observed pattern.	• *Sexual Arousal*: Occasional descriptions of sexual events without use of protection explained by being emotionally or physically “in the moment” without access to condoms. These events are consistently described as exceptions and rare.	
Kappa = .79	• *Communication about Risk*: No observed pattern.		• *General Attitudes about Risk and Typical Patterns of Risk Engagement and Protection*: Emphasize risk avoidance and need for protection across all kinds of events and circumstances. After occasional one-off unprotected events, return to pattern of risk avoidance and protection.
	• *Perceived Partner Characteristics:* Consistent use of protection with all partners. Discussion of avoiding having sex with partners perceived as engaging in risky behavior.		
	• *Relationship Trajectory and Risk:* Consistent condom use and use of other forms of protection even when having sex with long-term committed partners.		
**Risk reactors: medium risk, medium concern**	• *Emotional Factors*: Frequent use of protection with partners without an emotional bond. Change in use of protection after emotional bond developed for increased intimacy and developing pregnancy ambivalence.	• *Drug and Alcohol Use*: Occasional descriptions of unplanned unprotected sex with partners due to drinking or drug use. Occasional descriptions of unprotected sex events due to lack of concern about risk after substance use. Occasionally these events are first transitions from using condoms consistently to not using condoms at all with a partner. Once transition to unprotected sex, no pattern of drug and alcohol use affecting risk and protection decisions.	• *Concern about Pregnancy and STIs:* Some concern about pregnancy and STIs despite pattern of engaging in high risk behavior. Tendency to prioritize concern about either STIs or pregnancy while expressing lack of concern about the other issue. Describe being aware and in control of their risks because of a strategy for avoiding risk, such as having sex with a “safe” partner. STI testing (alone, as a pair, or exchanging papers) is often described as a technique for reducing risk and confirmation of “clean” status. Occasionally express a belief in sterility after not becoming pregnant after repeated unprotected sex.
N = 15 (10 female, 5 male)	• *Relationship Type*: Use of condoms and other projection with new partners, one-night stands, casual partners, etc. Pattern of no protection used with long-term committed partners who are considered “safe” because of an expectation of faithfulness.	• *Sexual Arousal*: Occasional descriptions of unplanned unprotected sexual events without use of protection due to being emotionally or physically “in the moment” and not wanting to reduce pleasure or feelings of intimacy with a partner. Occasionally these are events in which partners first transition from using condoms to not using condoms. Occasionally these events are first transitions from using condoms consistently to not using condoms at all with a partner. Once transition to unprotected sex, no pattern of sexual arousal affecting risk and protection decisions.	• *General Attitudes about Risk and Typical Patterns of Risk Engagement and Protection*: Tendency to stop using condoms early in relationships after developing emotional closeness to partner, becoming monogamous, and feeling that their partners were “safe”. Have “rules” for protection but also have rules for breaking rules (e.g. deciding partner is “safe”) or inconsistent application of rules.
Kappa = .67	• *Perceived Partner Preferences*: Occasional descriptions of use or lack of use of protection due to deferral to partner’s wishes.		
	• *Communication about Risk*: Frequent descriptions of unprotected events preceded by discussions of respondent and partner being “clean”. Sometimes discussions occur after unprotected sex.		
	• *Perceived Partner Characteristics:* Pattern of use of condoms with partners perceived as engaging in risk behavior or having unknown characteristics. Pattern of transition to unprotected sex with partners with increased familiarity.		
	• *Relationship Trajectory and Risk:* Frequent pattern of use of protection with new partners but eventually transitioning to consistent unprotected sex after a discussion of commitment or partners both being “clean”.

**Table 4:** **Exemplary quotations illustrating major themes and sub-themes**

**Relationship/Partner level**	
**Theme**	**Example quotes**
Emotional factors	• “*No, we haven’t really felt the need to (use condoms) since we got tested together and we both came back negative and neither one of us has cheated on each other so there’s no chance of either of us having anything.*” -- 18 year old female describing how developing trust between her and her partner led to unprotected sex
	• “*…we use condoms just because I'm not entirely sure where he's been.*” -- 21 year old female described how lack of trust of her partner influenced her to continue to use condoms with him
	• “*I was cool with her…I'd been with her for a while.*” -- 21 year old male describing how knowing his partner for a long time influenced a feeling of familiarity, leading to unprotected sex
	• “…*sometimes you get that feeling that the person – you kind of just know they’re the right person for you*.” – 18 year old female explaining not using condoms with her long-term partner with whom she developed an emotional bond
	• “*Because I think he sees me as a long-term relationship, and he wants to have kids already*.” – 18 year old female respondent describing how emotional closeness with her partner lead to unprotected sex in order to become pregnant
	• “*Like, we’re trying but we’re not. We’re like in the middle, you know?*” – 21 year old male describing how closeness to his partner lead to ambivalence towards pregnancy and, as a result, unprotected sex
Relationship type	• “*It was just a random person. I didn't know him*.” 21 year old female describing why she used a condom with a partner who was a stranger.
	• “*I don’t just want to go and fuck any random person…There’s too much risk, because I don’t know where they’ve been at and I don’t know their history and I don’t know what girls they’re being with. But he’ll sit there and tell me what girls he’s been with, who he’s been with and if I know them, then I know more of the background, disease-wise and even if we don’t use a condom, it’s like he knows he won’t bust in me because I told him I don’t want that shit*.” – 17 year old female describing her risk reduction strategy of having casual sex with a good friend rather than someone she does not know
	• “*Because he’s not my man, he’s not my boyfriend*.” – 14 year old female explaining why she uses condoms with a partner who is not her committed boyfriend
	• “*When I have a…very good girlfriend, very good woman, then yeah, we may not use a condom.*” – 20 year old male explaining that he would only have unprotected sex with a serious partner
Perceived partner preferences	• “*And then it kind of just became like, this one boy used them and this one preferred not to, so we didn't.*” – 22 year old female respondent describing how she relies on her partners to decide to use condoms or not
	• “*The only times I don’t (use condoms) is when she says not to, when she says she doesn’t want me to.*” – 21 year old male respondent describing how his use of condoms or not was due to his partner’s decisions
	• “*. . .she just offered the condom. She was like, ‘Here.’ I was like, ‘Alright.’ I have no problem with it. If anything, I think she’s helping me.*” -- 17 year old male respondent describing how using a condom at the insistence of his partner and how he appreciates her decision to use protection
	• “*Most of the time, we don't use protection. And I tell him not to cum inside of me, but he'll still do it. And I've tried using condoms, but he won't let me.*” –17 year old female explaining that she wants to use condoms but her boyfriend does not want to use them and does not use withdrawal even though she asks him to do that.
Communication about Risk	• *“If they stutter or think about it for a minute, I’d just be like, ‘All right, never mind.*’” – 23 year old male describing his strategy for determining partner riskiness by asking them if they have an STD and evaluating their reaction
	• *“I asked him if he was clean, and he said that he had gotten tested and he seemed like a real person that wouldn't really lie to you.*” – 22 year old female describing the reassurance she received from her partner about his lack of an STD
	• “*I already knew she didn't have anything…I'd seen her paperwork*.” – 22 year old male describing his confidence that his partner did not have an STD because she presented him with results of an STD test
	• *“It was more just like an offhand…hey…you don’t have anything, right? Are we still going to use these?”* -- 22 year old female respondent describing her discussion of condoms with her partner prior to engaging in unprotected sex
	• “*She said that she was clean…Thank you, God, I didn’t catch nothing*.” --22-year-old male respondent describing his reaction after a family member of a woman with whom he had unprotected sex told him she was HIV+
	• “*It’s the papers that prove it*.” – 18 year old female describing her attitude towards the need to see documentation from her partners about their STD status to decide to have unprotected sex or not
	• “*Are you clean? I can prove it. Look.*” 20-year-old male who is tested every 3 months describes how he shows his test papers to each of his new partners and asks for theirs prior to unprotected sex
	• “*Of course we asked the question, ‘Are you safe?’…And he's, like, I'm safe, you know, are you safe? We kind of took our word for it and then we later got tested and found out we're both safe*.” – 22 year old female describing communication with her partner prior to engaging in unprotected sex for the first time
	• “*I told him this was the ninetieth day so, for me, I'm off birth control so you need to start using a condom or pull out*.” -- 18 year old female described what she told her partner about protection after her Depo-Provera shot wore off
	• “*I was like…‘You can get me pregnant.’ And he was like, ‘I’m not gonna nut in you*.’” -- 14 year old female describing her conversation with her partner prior to having unprotected sex after they ran out of condoms.
	• “*He says it feels better when the guy comes inside the girl, so when he wants to come inside me, he’ll put a condom on, so he can cum in the condom*.” – 17 year old female describing communication with her partner over using a condom or engaging in withdrawal
	• *I just automatically do that shit…it just happens.”* -- 20 year old male respondent explaining why he did not have a conversation with his partner about using a condom
Perceived partner characteristics	• “*For the most part, I trust him. But he's also known as a ‘man whore’…And I was pretty concerned about where he had been*.” –21 year-old female respondent explaining that she used condoms with her partner because of his reputation
	• “*There’s only three dudes she slept with in her whole life, and I just happened to know all of them*.” -- 21-year-old male respondent explaining his lack of concern about not using condoms with his partner because he knew her sex history
	• “*If I’m paying like $30 for a hooker then, yes, I’m going to use a condom.*” – 20 year-old male respondent who frequently had unprotected sex said that he would not have unprotected sex with a prostitute
	• “*I know that the women that I mess with…I make sure… I ask them…or check with the homies and shit like that if not.*” – 22 year-old male describing a conversations with his friends about a potential sex partner prior to unprotected sex
	• “*I caught chlamydia twice and it came from really hot girls, both times….I get a little bit uglier girls, I been clean every time.*” – 21 year old male explaining how he now engages in unprotected sex with ugly girls because they are less risky
Relationship trajectory and risk	• “*I started getting more sensual…with our lovemaking… around that time it was around Valentine's Day and since we had been hooking up and seeing each other for a while, like, everything was a little bit more passionate when I tried to approach her*.” 22-year-old male said that he and his partner transitioned from consistent condom use to unprotected sex when their sexual relationship became more passionate
	• “*It was just kind of like they were getting in the way… They would slip off and then you'd have to put it back on again and then sometimes they'd get stuck inside me and I'm just, like, oh my God.*” – 23 year-old female explaining why she and her boyfriend transitioned from always using condoms to never using them
	• *“We just kind of stopped using them…We just kind of stopped randomly.”* – 22 year old male describing how he and his partner transitioned from using condoms consistently to not using them at all for no particular reason
	• *“Yeah, just like ran out and then it was like, oh, fuck it. Didn't care.”*—20 year-old male describing the event that lead to transitioning from consistent condom use to unprotected sex with his partner because he ran out of condoms and was too “lazy” to go get a new one and did not care about the consequences
	• “*I run up there and I grab a condom and I come back and I tried and she tried, too. She said, ‘It’s not working.” I was like, ‘Nope,’ and I threw the condom. And she’s like, ‘Oh well, at least you tried to get a condom.*’” – 21 year old male describing he and his partner’s attempt to use a condom one time when they first had sex prior to engaging in consistent unprotected sex
	• “*I guess we were just like really into it, and I didn’t want one on him because I just wanted to like get the feeling better…It doesn’t feel as pleasurable with it on as it does with it off*.” – 18 year old female respondent describing an isolated incident of unprotected sex with her partner with whom she typically had protected sex
	• “*We just wouldn’t have sex*.” 22-year-old male who said he consistently used condoms answering a question about what he would do if he and his partner ran out of condoms.
**Event circumstances level**	
**Theme**	**Example quotes**
Drug and alcohol use	• “*We were drunk, and we were just like in the moment, you know?*”—22 year-old female describing why she had unprotected sex with her partner during one event
	• “*Under the influence or not, I’m still aware of what I’m doing…I know that if I have sex without a condom, there’s still a really good chance that I could end up with an STD or a kid…no matter how drunk I am.*”—16 year old male arguing that being drunk does not prevent him from knowing that he needs to use a condom to avoid risk
	• “*When people tell me, ‘Oh I got drunk. I didn’t mean to do it.*’ I’m just like there’s no way.” – 18 year old female describing her attitude towards people who blame having unprotected sex on being intoxicated.
Sexual arousal	• “*Yeah, at the moment, I just didn’t care. I didn’t care what happened, the only thing I was focused on was bed, naked woman, me, that’s it, you know?*” 16 year old male respondent explaining why he atypically did not use a condom
	• “*…because it was right then and there and she was asking for it so…*” -- 21 year old male describing how the sexual arousal of his partner led him to engage in sex without condoms
	• “*I was into it too much, so it happened.*” -- One 21 year old male respondent said that he did not pull out because he was too sexually aroused and was concerned about pregnancy afterwards
**Individual level**	
**Theme**	**Example quotes**
Concern about pregnancy and STIs	• “*I have to be in a situation where I feel like I can actually take care of myself, my girl, and my child. And right now we’re not at that level yet.*” 22-year-old male describing his use of condoms because of his concern about pregnancy because he was not stable enough in his life to become a parent
	• “*I am struggling to take care of myself and how the fuck am I going to take care of an infant?*” – 21 year old female discussing her concern about becoming pregnant and why she does not have unprotected sex
	• “*I'm just concerned with every STD. I just wouldn't want to get one. It would be horrible, because you see people suffering with it. And I would just really be ashamed of myself.*” – 15 year old female discussing her desire to protect herself because of her concern about getting an STI
	• “*Because in the past…I have…not used protections and came, but they never seem to get pregnant. So…I kind of don't worry about it so much*.” – 22 year old male describing his lack of concern about engaging in unprotected sex
	• “*Didn’t care. . .life as we know, it’s over*.” –18 year-old male’s description of why he often had unprotected sex instead of protecting himself against STIs
	• “*…you’re just taking the pill for nothing. If God wants you to have a baby, He’ll let you have a baby. If He doesn’t, He won’t let the miracle happen. That’s how I see it. God knows when you’re ready for a baby and when you’re not.*” –22 year old female explaining why she does not use contraception and is not concerned about pregnancy
General attitudes about risk and typical patterns of risk engagement and protection	• “*Hey, if we’re going to have sex, you need to show me some papers if you want to do it without a condom.*” -- 14-year-old female described her typical interaction with partners who wanted to have unprotected sex
	• “*I don’t let them put it in without a condom on…I think guys should always use condoms because it like reduces like spread of whatever. And it makes the people a whole lot safer*.” – 21 year old female discussing her consistent approach to asking all partners to use condoms
	• “*I'm really all about safety. I'm just a freak about that.*” -- 15 year old female discussing the intensity of her concern about preventing risk
	• *“I just automatically do that shit…it just happens.”* -- 20 year old male respondent explaining his automatic use of condoms with all partners
	• “*For me it’s just like the first time rule kinda, you’ve kinda got to put it on.*” -- 18 year old female explaining her typical pattern of initially using condoms with new partners but not after the first time
	• *“I usually use them with like new partners…I think I’ve used them with every new partner…usually the first time, or the first few times, but then after that, like, I just sort of stop.”* – 22 year old female describing the typical pattern of starting out using condoms with partner but then just stopping using them after the first sex or first several sexual encounters
	• “*Because I don't like them…Because they'll break. They break! So, they break…you still get pregnant. What's the difference?* – 22 year old female explaining that she has never uses condoms because they are unreliable
	• “*I would never get off with a condom…Because you get no feeling…I ruined it the first time I had sex without a condom.”* –21 year old male explaining that he has never used a condom because they inhibit sexual pleasure

### Relationship/Partner-level influences on unprotected sex

#### Emotional factors

Respondents frequently discussed emotional aspects of their relationships that contributed to their decisions to use or not use condoms. Sixteen respondents (43%) made explicit statements about how emotional factors lead to decisions about unprotected sex with particular partners. These emotional factors included statements related to trust, familiarity with the partner, and developing an emotional connection with the partner. Respondents who described trust as a factor in having or not having unprotected sex typically stated that they were in a monogamous relationship with their partner and they were not concerned about contracting STIs because they trusted that the partner was not having sex with other people. For example, an 18 year old female respondent said, “*No, we haven’t really felt the need to (use condoms) since we got tested together and we both came back negative and neither one of us has cheated on each other so there’s no chance of either of us having anything.*” Although trust was usually linked to confidence in sexual fidelity, there were also examples of respondents who linked trust to unprotected sex even when they knew their partners had sex with other people during their relationship. Several of these respondents said that they trusted their partners to use condoms when they had sex with these other partners and, therefore, did not feel the need to use condoms. On the other hand, lack of trust was mentioned as a reason for using protection. Several respondents described condom use when they suspected or had knowledge that their partner had been having sex with other people or were not certain about their partner’s fidelity. In addition to citing feelings of trust specifically, respondents often referred to general feelings of familiarity and emotional closeness to their partners as reasons for not using condoms. Respondents who were having unprotected sex also sometimes explained that they were not using condoms because of a desire for pregnancy with their partners or because they were ambivalent about pregnancy. For example, a 21 year old male respondent said, “*Like, we’re trying but we’re not. We’re like in the middle, you know?*”

#### Relationship type

Besides the emotions towards their partners, respondents described reasons for engaging in protected or unprotected sex based on how they classified their relationship. Respondents usually identified a category into which their relationship fit, such as committed/serious relationships (steady boyfriends/girlfriends or fiancés), casual relationships (frequent sex partners who are mostly friends or “friends with benefits”), or one-time-only sexual relationships (either strangers or “one-night-stands”). Twenty-three respondents (62%) mentioned having committed relationships, 18 mentioned casual relationships (48%), and 11 respondents mentioned having one-time-only sexual relationships (30%). Respondents sometimes described not using condoms because their relationship fell into the committed category, or described using condoms because the relationship was not committed (casual or one-time-only). For example, a 14 year old female respondent said that she used condoms with her partner, “…*because he’s not my man, he’s not my boyfriend*.” To explain the circumstances when he might not use condoms, a 20 year old male respondent said, “*When I have a…very good girlfriend…then yeah, we may not use a condom.*” However, respondents also described relationships that were exceptions to these patterns; several respondents described using condoms consistently with their committed partners (primarily out of concern over pregnancy) whereas others described engaging in unprotected sex with casual partners/strangers.

#### Perceived partner preferences

In addition to their motivations to use or not use condoms with partners because of their emotional connections to these partners, respondents also described decisions about protection that were based on the desires of their partners. Respondents gave several examples of sexual events when they either did or did not use a condom based solely on their partner’s preference. For example, both male and female respondents described instances when their partners insisted on condom use. For example, a 21 year old male respondent said, “*The only times I don’t (use condoms) is when she says not to, when she says she doesn’t want me to.*” On the other hand, three female respondents described examples of unwanted unprotected sex in abusive relationships. Other respondents described examples of their partners engaging in some form of deception that lead to increased risk, such as lying about intentions to “pull out”, secretly tampering with contraception methods to increase the chances of pregnancy, or a partner not revealing that he/she had an STI in order to have unprotected sex. For example, a 17 year old female respondent said, “*And I tell him not to cum inside of me, but he'll still do it.”*

#### Communication about risk

In addition to discussing condoms, respondents also described other examples of communicating with partners about protection, STIs, and contraception prior to making decisions about protection. Fourteen respondents (38%) mentioned that they had discussions with their partners about protected sex, STIs, or whether they or their partners were “clean” prior to engaging in unprotected sex. For example, a 22 year old female respondent said, “*I asked him if he was clean, and he said that he had gotten tested and he seemed like a real person that wouldn't really lie to you.*” Additionally, 9 respondents (24%) described discussions about contraception methods besides condoms. Occasionally, respondents discussed how these conversations lead to condom use or not engaging in sex. However, for the most part, these discussions preceded unprotected sexual events. Some respondents asked particular partners to show them paperwork that demonstrated that they had a recent negative test for STIs. Other respondents said that they did not have to see the paperwork and relied on their partners to tell the truth about their STI status. In contrast, other respondents stated that they never had sex without seeing STI paperwork confirming no infection. For example, an 18 year old female respondent said, “*It’s the papers that prove it*.” Respondents discussed getting tested with their partners to confirm that neither of them was infected. Sometimes partners got tested together prior to engaging in unprotected sex and other times they got tested together after already engaging in unprotected sex.

Frequently, respondents described events in which they negotiated with their partners about using the withdrawal method (“pulling out”) for avoiding pregnancy prior to having unprotected sex. Fifteen respondents (41%) described at least one sex event in which the withdrawal method was used. Several respondents characterized using the withdrawal method as being “careful.” Often, male partners would promise to not “cum” or “nut” inside their female partners in exchange for not using condoms. Sometimes female respondents described events in which they told their male partners to pull out because they were not using contraception. Several respondents discussed events in which the male partner promised to pull out but did not. These events were typically described as accidental. In contrast, several male respondents described typically engaging in withdrawal but occasionally deciding to use condoms prior to having sex in order to ejaculate without having to pull out.

On the other hand, seven respondents (19%) also described events with partners in which there was no communication with their partners about protection. These respondents either stated explicitly that they had no prior discussion about condoms before using them or suggested that they did not need to discuss condom use because it was simply “known” that condoms would be used. For example, a 20 year old male respondent said, *“I just automatically do that shit…it just happens.”* Respondents also frequently described events in which the male partner pulled out without any prior discussion. Some respondents said that they engaged in unprotected sex without any prior discussion with their partners about risk or protection because they did not consider their partner a risk.

#### Perceived partner characteristics

In addition to their relationships with partners, respondents also described making decisions about having unprotected sex based on characteristics of the partners. Several respondents mentioned that the reputation of the respondent was a factor in deciding to use or not use condoms. Other respondents also described being concerned about having unprotected sex with partners based on their physical appearance (e.g., appearing “unhygienic,” having “track marks” in their arms from needles, having too many tattoos), or their behavior (e.g., being too willing to engage in unprotected sex). Respondents stated that they did not engage in unprotected sex with certain types of partners they associated with more risk, such as “gutter punks” or prostitutes. Fifteen respondents (41%) described evaluating their partners’ physical appearances, personalities, reputations or behavior characteristics to decide if they should engage in unprotected sex. For example, one 21 year old male respondent, who explained that he had heightened concern about getting an STI from attractive partners, said his strategy was to, “…*get a little bit uglier girls”.*

#### Relationship trajectory and risk

Comparison of respondents’ descriptions of multiple sex events with particular partners revealed different patterns of condom use within particular relationships. One major pattern was consistency either in use of or non-use of condoms. Fourteen respondents (38%) described relationships in which they consistently used condoms with partners. Some of these relationships were long-term partnerships whereas others were casual or one-time-only sexual relationships in which respondents never considered not using a condom. Several respondents described relationships in which they consistently used condoms but had one or two exceptions in which they engaged in unprotected sex. These exceptions typically coincided with times that they were either intoxicated or were sexually excited but did not have access to condoms or were rushed. In contrast to these respondents who described relationships in which they always or nearly always used condoms, 14 respondents (38%) had relationships in which they never used condoms. Six of these respondents (16%) said that they were not concerned about pregnancy or STIs, sometimes despite having had a past experience with STIs and/or pregnancy. Thirteen respondents (35%) said that they “knew” that their partners were “clean.” Others were using some other form of birth control and did not feel the need to use condoms. Some respondents used condoms in the past or with other partners but never used them with certain partners. Seven respondents (19%) said that they had never used condoms with any partner. Although most of the respondents’ consistent unprotected sex partners were long-term partners, a few were one-time-only sex partners.

Another pattern was inconsistency in condom use throughout a relationship. Fourteen respondents (38%) described patterns with their partners in which condom use fluctuated over the course of their relationships. Many of these relationships involved consistent condom use early on in the relationship which transitioned into not using condoms. In these cases, respondents often explained that they stopped using condoms with their partners because of the emotions they felt towards their partner, such as becoming more familiar with them or feeling a growing sense of trust or emotional attachment to the partner and a desire to increase their intimacy through unprotected sex. For example, a 22 year old male respondent said, “*I started getting more sensual…with our lovemaking”* A few respondents said that the growing emotional attachment to their partners lead them to stop using condoms because they wanted to have a baby with their partner. For some of the respondents, the transition to unprotected sex began during an event in which they had unplanned unprotected sex because they were high or intoxicated, or because they did not have condoms and were too sexually aroused to postpone sex. Other respondents described how unprotected sex started because condoms were “getting in the way” with their lovemaking experience because they made sex less pleasurable, caused the male partner to lose his erection, caused an allergic reaction (due to latex), or the condoms fell off. Three female respondents (8%) and two male respondents (5%) described unprotected sex events due to male partners having problems using condoms and maintaining an erection.

A distinct pattern within the inconsistent condom use relationships was that some respondents transitioned from using condoms to not using condoms once they had unprotected sex for the first time. For example, a 20 year old male respondent said, *“Yeah, just like ran out and then it was like, oh, fuck it. Didn't care.”* Several respondents described having conversations with their partners about STIs or pregnancy prior to permanently transitioning away from using condoms. Frequently, the transition to unprotected sex happened soon into the relationship. Seven respondents (19%) described using condoms during the first few times they had sex with a partner but then never using condoms again. Some respondents explained that this was because they had a “rule” for not engaging in unprotected sex immediately with a partner. A few respondents described experimenting with condoms the first time they had sex with a partner but then deciding to have unprotected sex.

### Event influences on unprotected sex (within-relationship)

#### Drug and alcohol use

When discussing particular relationships, respondents also discussed circumstances of particular events that contributed to their behavior deviating from their typical patterns of protection. The most commonly cited event circumstance that led to use or non-use of protection was drug and alcohol use of the respondent and/or the partner. Twenty-one respondents (57%) described at least one sexual event in which drugs and/or alcohol had made them more likely to forego condom use and ten respondents said that drugs/alcohol lead them to engage in other types of risky sex encounters. Many respondents stated explicitly that their use of alcohol or drugs made them more likely to engage in unprotected sex. A typical comment (from a 21 year old female respondent) was, “*We were drunk, and we were just like in the moment, you know?*” On the other hand, a smaller number of respondents said that their use of drugs and/or alcohol was unrelated to their non-use of protection and did not believe others when they blamed unprotected sex on substance use. For example, a 16 year old male respondent said, “*Under the influence or not, I’m still aware of what I’m doing.”* Eighteen respondents (49%) described sexual events in which they used alcohol and/or drugs and also used a condom. Two participants (5%) said that they were more likely to use condoms when intoxicated.

#### Sexual arousal

The second most prominent theme related to respondents’ descriptions of deviating from their typical pattern of unprotected sex during one event was the impact of feelings of intense sexual arousal. Respondents described how they engaged in unprotected sex uncharacteristically during certain events because they were overwhelmed by these feelings. Five respondents (14%) said that they had unprotected sex because they were not thinking about risk “in the moment” and 4 respondents (11%) said that they had unprotected sex because they wanted to experience greater pleasure at the time. For example, a 21 year old male respondent said, “*I was into it too much, so it happened.*” Seven respondents described events in which they did not have condoms available and decided to have unprotected sex because they did not want to wait. On the contrary, a few other respondents described events in which they did not engage in unprotected sex even though they were aroused and condoms were not available.

### Individual influences on unprotected sex (across-relationship)

#### Concern about pregnancy and STIs

Comparisons of the multiple relationships described for each respondent revealed several different patterns of protection and concern about risk that were not influenced by relationship characteristics or circumstances of particular sex events. Two primary individual respondent characteristics that were repeatedly linked to protected or unprotected sex were concerns about STIs and concerns about pregnancy. Although the majority of respondents cited concerns about both pregnancy and becoming infected with an STI as reasons for engaging in protective behavior, there were exceptions and some variation in how respondents expressed their concerns about pregnancy and STIs. Eight respondents (22%) used both condoms and other forms of contraception because they were concerned about both pregnancy and STIs and wanted to do everything possible to prevent pregnancy and STIs. Other respondents were primarily concerned about one or the other. Eight respondents (22%) said that they were more concerned about pregnancy than getting an STI. Seven of these respondents (19%) said that they did not bother to use condoms because they were using other methods of pregnancy prevention, such as oral contraception or a Depo-Provera shot, which made using condoms unnecessary. On the other hand, 11 respondents (30%) said that they were more concerned with STIs than pregnancy.

Respondents expressed a range of reasons for being concerned or not concerned about pregnancy. The most common explanation for concern about pregnancy was that they were not financially stable enough to take care of a child and had other goals (e.g. continuing their education) that would be compromised if they had a child. For example, a 21 year old female respondent said, “*I am struggling to take care of myself and how the fuck am I going to take care of an infant?*” Nine respondents (24%) had already experienced pregnancy in the past including one respondent who was pregnant at the time of the interview. Some respondents referred to these previous pregnancies as explanations for their motivation to use protection. Six respondents (16%) stated that they used condoms because they were concerned about pregnancy while also stating that they had a desire to have a child in the future.

In contrast, ten respondents (27%) said that they were unconcerned about the risk of pregnancy when engaging in unprotected sex. Five of these respondents (14%) said that they were unconcerned because they desired a pregnancy. Other respondents who did not want a baby said that they were unconcerned about pregnancy because they thought that they were sterile and could not have children. Six respondents (16%) expressed some belief in their own sterility either currently or in the past. Several respondents said that they believed they were unable to become pregnant or impregnate a partner because they have had a lot of unprotected sex either with one or multiple partners and none of these unprotected events resulted in a pregnancy. On the other hand, 4 respondents said that they were unconcerned because they thought that there was nothing they could personally do to prevent a pregnancy because it was inevitable. For example, a 22 year old female respondent said, “*If God wants you to have a baby, He’ll let you have a baby”.*

Similar to pregnancy, most respondents expressed a desire to engage in protected sex to avoid contracting an STI. Most respondents who were concerned about becoming infected with an STI were non-specific about which STI most concerned them. Several respondents had previous experience with various types of STIs (3 respondents (8%) or their partners had herpes, 1 (3%) was HIV+, and 7 (19%) had some other form of STI) and these experiences influenced their motivation to protect themselves. Others discussed times they were tested for an STI after engaging in unprotected sex and worrying they might be infected. Seventeen respondents (46%) discussed either getting an STI test after an event that made them concerned about being infected or being in the habit of getting tested for an STI regularly. Several respondents described getting tested to confirm that they were not infected after engaging in unprotected sex. Respondents who were tested regularly (typically every 2 to 3 months or so) used the documentation of their STI test to facilitate sexual encounters. Several respondents who did not do anything to prevent either pregnancy or STIs nevertheless got tested regularly. Other respondents who consistently used condoms also got tested regularly because of their concern about STIs.

Although many respondents were concerned about STIs, eight respondents (22%) who engaged in unprotected sex expressed little to no concern about STIs. Some of these respondents were not concerned because they believed that they were in monogamous relationships with “clean” partners or relationships with partners who protected themselves when they had sex with other partners. When asked why they did not protect themselves against STIs, several respondents replied that they did not care about protecting themselves. Some of these respondents said that they thought getting an STI was inevitable and some said that their lives were already as bad as they can be and they were not motivated to protect themselves.

#### General attitudes and typical patterns

Other individual characteristics that affected unprotected sex included general attitudes about condoms, negative reactions to various methods of protection, and typical patterns of protection that respondents engaged in across their relationships. Some respondents who expressed very positive views of protection and condoms said that they used them consistently across their relationships and carried them all the time. For example, a 21 year old female respondent said, “*I don’t let them put it in without a condom on*.” Some respondents described their typical patterns of condom use that were conditional on circumstances such as always using condoms the first time they had sex with a new partner (but did not necessarily use them the second time). For example, a 22 year old female respondent said, “*For me it’s just like the first time rule kinda, you’ve kinda got to put it on.*” On the other hand, some said that they had never used condoms and never planned on using them or had tried them and would not use them again because they did not like them.

### Respondent risk/protection profiles

Table 3 presents the three risk/protection profiles that emerged from pile sorting analysis of the within-respondent theme summaries described in the Qualitative Data Coding section. This table also presents descriptions of the pattern of each of the above sub-themes within each of the risk/protection profiles. The three patterns reflected different approaches to risk behavior and concern about risk. The three patterns that emerged were 1) Risk Takers: *high risk, low concern*, 2) Risk Avoiders: *low risk, high concern*, and 3) Risk Reactors: *medium risk, medium concern*. The Risk Takers (N = 12, 32%; 7 female, 5 male) described the most overt risky behaviors. As the sub-theme pattern descriptions in Table 3 demonstrate, the primary driver of risk evaluation for these respondents is located in the individual level. These respondents expressed the least amount of concern about STIs and pregnancy and tended to describe typical patterns of risky behavior. Sub-themes at the relationship/partner- and event-level for this group did not appear to have any association with their decisions about risk and protection. Respondents in this group tended to engage in risky behavior regardless of relationship/partner or event circumstances. Respondents in this category are best represented by Example 1 in Figure 1. In contrast, the Risk Avoiders group (N = 10, 27%; 3 female, 7 male) engaged in high risk sexual behavior rarely, expressed strong concerns about consequences of risk behavior, and described their typical patterns and personal rules of behavior that the respondents followed to avoid risk. Similar to the Risk Takers group, the relationship/partner and event-level sub-themes for the risk avoider did not demonstrate a pattern of association with their decisions about risk and protection. Respondents in this group tended to avoid risk behaviors regardless of relationship/partner and event circumstances. Respondents in this category are best represented by Example 2 in Figure 1.

The final row of Table 3 describes the sub-themes associated with the Risk Reactors profile (N = 15, 41%; 10 female, 5 male). In general, respondents in this group exhibited a mixture of examples of risk and protective behaviors. They expressed less consistency in their expressions of concerns about risk and described inconsistent and contradictory patterns of protective behavior to avoid risk. Unlike the two previous groups, the Risk Reactors often described situations in which they engaged in unprotected sex in reaction to one of the relationship/partner or event circumstance level sub-themes. Examples 3 and 4 in Figure 1 represent two hypothetical individuals who would best be classified into this category.

## Discussion

This study offers the most extensive qualitative exploration to date of the patterns of risky sex and protection among homeless youth – in particular the patterns of unprotected sex in their romantic relationships. Similar to other studies of unprotected sex among homeless youth, we found that multiple intersecting factors influenced their risk behavior including characteristics of their relationships, their partners, communication about risk and protection, attitudes about protection and contraception, concerns about HIV/STIs and pregnancy, and temporary circumstances such as their level of intoxication. We found that these different levels of influence often interacted with each other in complicated ways to make risky or protective behaviors more or less likely.

The primary goal of this project was to illuminate the role of relationships in the decision about whether to use condoms among homeless youth and to explore how relationship-level factors intersected with factors at other levels of influence. We also aimed to inform the development of condom use interventions customized for issues faced by homeless youth. We found support for our argument that a multi-level intervention informed by an ecological approach would be the most promising approach to encourage condom use among homeless youth. We also found support that these interventions should include a focus on the relationship context of risk. Similar to other homeless populations [28-30], respondents described decisions about condom use relevant to their feelings about their partners and the trajectory of their relationships with them. Youth often used feelings of closeness and safety and beliefs in how “clean” their partners were to make decisions about use or non-use of condoms. Negative emotions towards partners, such as lack of trust, also appeared to play a role in condom use decisions. Even respondents who engaged in risky sex on a consistent basis sometimes described using condoms with partners they considered to be riskier than themselves.

Exploration of the association between relationship trajectories and condom use reinforced previous studies’ conclusions that condoms become less likely in relationships once partners develop strong emotional bonds [23,42-47]. The strengthening of an emotional bond between partners appears to trigger a process in which the likelihood of unprotected sex increases: as the emotional connection grows stronger, trust increases, feelings of exclusivity in the sexual relationship grow - which in turn lowers the concern about becoming infected with an STI. At the same time, feelings of commitment increase which lowers the concern about pregnancy or even makes pregnancy desirable. However, the timing and pace of this process appears to differ considerably across respondents. Some respondents described this process as taking place over a long period of time while for others it was almost immediate. Several respondents even appeared to experience this transition after having had sex with their partners only once.

Similar to another study of unprotected sex among adolescents [48], we did not find strong evidence that the development of condom use interventions that explicitly target gender roles and power dynamics would be effective for homeless youth. Respondents did occasionally discuss deferring to their partners to make decisions about protection. Also, a few female respondents discussed being pressured to engage in unprotected sex by their male partners. However, this circumstance was relatively rare and a few males also referred to being pressured by their female partners to engage in unprotected sex. Most respondents who discussed deferring to their partners appeared to do so willingly. Also, some discussed engaging in protective behavior at the insistence of their (male and female) partners. This suggests that gender based interventions, while successful with some populations in different cultural or socioeconomic settings, may not be the most effective approach with homeless youth.

Our findings also add nuance to our understanding of the role of communication about risk and condom negotiation prior to use of protection during sexual activity [49]. We found that different types of communication can lead to different condom use outcomes. In some examples, discussion of risk led to greater protection decisions while in other examples couples used discussion to convince themselves (perhaps erroneously) that having sex without a condom was not a risk. In other instances, couples did not discuss condom use because they had previously decided (explicitly or implicitly) that they would use or not use condoms with each other. We also have examples of condom use due to a request from a respondent or their partner while we also found a few examples of these requests being ignored by partners. Therefore, our findings suggest that interventions targeting greater communication about protection and condoms should take into consideration the relationship context of this communication and promote the type of communication that will most likely trigger greater protection.

While relationships often played a central role in the youths’ discussions of their sexual experiences, they also discussed other factors that influenced their unprotected sex. While some youth made decisions to have isolated unprotected sex experiences because of temporary circumstances, other youth engaged in protective or unprotected sex regularly regardless of their relationships or the circumstances at the time they decided to have sex. Respondents even disagreed about whether excessive drinking and drug use contributed to having unprotected sex. Although substance use was often identified as the main cause of risky behavior, others said that they believed that alcohol or drugs were sometimes used as an excuse for engaging in unsafe behavior.

Perhaps the most novel finding of this study is the identification of different types of homeless youth with different risk profiles. This finding has important implications for intervention development. Youth in these three risk profiles differed from each other through different combinations of individual characteristics, relationships, and tendencies towards types of risky sexual events. This finding supports other studies that identified patterns of heightened risk characteristics among certain homeless youth [50]. We found that just under one-third of the youth engaged in risky behavior regularly and did not show concern about the consequences or demonstrate any motivation to change their behaviors. We also found that nearly one-third of the youth exhibited the opposite approach to risk. These youth not only minimized their risky sexual behavior by using (sometimes multiple) effective protection strategies but they often described their concern about the consequences of their few risky sexual experiences.

The youth who did not fit easily into either of these categories formed the largest and most diverse category. These youth often stated concerns about risk, but their protective behaviors were often inconsistent and relied on ineffective methods such as withdrawal, asking their partners if they were “clean” or getting regular tests for STIs while continuing to engage in risky sexual behavior. It is possible that, because these youth had not made a choice to either actively avoid all risk or to be completely unconcerned about the consequences of risk behaviors, their behavior was inconsistent because they were more affected by the context of their sexual events or the trajectories of their relationships than the other two types of youth. It is also possible that, although these youth expressed concern about the consequences of STIs or pregnancy, they chose protective behaviors not because of their effectiveness in reducing risk as much as their effectiveness in reducing concern about risk.

This interpretation is consistent with another study’s finding that homeless youth who discussed HIV risk with their partners were less likely to feel regret about engaging in unprotected sex with these partners [25]. This finding strongly suggested that the content of the discussion about HIV was directed at lowering the concern about HIV rather than increasing awareness of HIV as a risk. Many of the discussions of respondents in this study support this interpretation. Therefore, although these youth demonstrate a pattern of risky behavior and describe their concerns about exposing themselves to risk, their common protection strategies (pulling out, going for an STI test after engaging in unprotected sex, hearing their partner say that he/she is “clean” without confirmation, etc.) may serve to make them feel better about risky behaviors that they do not intend to change. Each of these methods requires low effort and does not require delaying pleasurable experiences. Rather than being strategies to actively reduce risk, they may be strategies to reduce their cognitive dissonance about engaging in behaviors that they know have immediate positive consequences while also having potential negative long-term consequences.

The conceptualization of three risk profiles of homeless youth also helps to explain other findings from previous research. The three different risk/protection profiles explain why different factors are associated with unprotected sex in multi-level findings controlling for each other [25]. Decisions about condom use for the two extreme risk/concern groups appear to be more influenced by their individual characteristics than their relationships. On the other hand, the middle risk/protection group appears to be more at risk due to relationship and event circumstances. Therefore, individual tendencies or relationship characteristics may be more or less important depending on the individual homeless youth. These findings also help explain seemingly contradictory findings about the association between drugs and alcohol and unprotected sex in multi-level dyadic analyses (which found no association between a pattern of drug and alcohol use during sex and condom use with a particular partner) [25] and event-level analyses (which found that drug use preceding sexual events made condom use less likely) [12]. The present study’s findings demonstrate that, although substance use is a barrier to condom use during particular sex events for all youth, youth who commit to either using or not using condoms consistently were less likely to change their typical behavior and were better able to overcome impairment in their judgment due to substance use.

Collectively, these findings suggest that interventions promoting safer sex practices among homeless youth will need to take an ecological approach and account for these multiple pathways to risk [15]. Ecological approaches recognize that intervening on specific behaviors requires addressing the multiple overlapping environments (e.g. individual, social, cultural, organizational, physical) in which behaviors are nested. Intervention approaches that are successful at reducing unprotected sex with one type of homeless youth may have no effect on condom use with another type of youth. For example, an approach that uses a motivational interviewing (MI) intervention style may be appropriate for youth in the medium or high concern category because these youth exhibit some inherent motivation to avoid risk [51]. Youth in the medium concern category may benefit from a neutral MI style intervention that helps them recognize on their own how their risk prevention strategies are not consistent with their concerns about risk. Those in the high concern category may benefit from a more focused development of strategies to avoid the circumstances that lead to their infrequent high risk experiences. However, those in the high risk/low concern group would not likely benefit for an MI approach because of their apparent lack of motivation to avoid risk. These high risk youth might require more extensive interventions into significant mental health or substance use problems and their behavior and lack of concern about their futures may be the result of prior traumatic experiences that cannot be easily addressed with brief interventions. Previous studies of unprotected sex among homeless youth and homeless women have demonstrated that experiences of childhood abuse increase the odds of engaging in unprotected sex in particular relationships [25,26] and may lead to different risk profiles [52].

The aim of this study was to explore the complicated process of unprotected sex among homeless youth in order to inform theory and policy. This study does have several limitations worth noting. The aim of the study was to generate theory based on empirical data rather than to test for statistically significant associations or to estimate prevalence of any characteristic or behavior in a larger population. The identification of categories of youth and description of thematic patterns in their descriptions of unprotected sex events and sexual relationships cannot be immediately generalized to all homeless youth or even all homeless youth in Los Angeles. Although we made efforts to randomly recruit and select youth from a variety of locations in Los Angeles, our sample size is too small to enable estimates of the characteristics of any particular population. Thematic findings, including the identification of types of homeless youth and their relative size, should be considered descriptive of the current sample. We also do not know if the themes and patterns in risk profile that we identified are unique to homeless youth because we had no comparison group. It is possible that we identified patterns that are also relevant for other youth who are not homeless. Studies using more standardized data collection methods with a larger, representative sample and comparison samples are required to produce estimates of population parameters and test hypotheses generated in this study.

## Conclusions

This study suggests that romantic and sexual relationships play a central role in many homeless youths’ decisions regarding unprotected sex. The relationship context of unprotected sex has received relatively less attention than other factors, such as education about condoms and STI risk or substance use and risky sex. Condom promotion interventions should focus on increasing awareness of how relationship trajectories can increase the likelihood of unprotected sex and expose homeless youth to the risk of pregnancy, STIs and HIV. We also found that for many youth the relationship context was less important because they were strongly committed to a pattern of risk behavior or risk avoidance regardless of their relationships. We recommend interventions that comprehensively address these multiple interacting factors in order to provide the greatest likelihood of reducing risk behavior among homeless youth and improving their sexual and reproductive health.

## Abbreviations

HIV: Human immunodeficiency virus
STIs: Sexually transmitted infections
MI: Motivational interviewing
HSPC: Human Subjects Protection Committee

## References

[1] Rotheram-Borus MJ, Song M, Gwadz MV, Lee M, VanRossem R, Koopman C (2003). Reductions in HIV risk among runaway youth. Prev Sci.

[2] Rotheram-Borus MJ, O'Keefe Z, Kracker R, Foo H-H (2000). Prevention of HIV Among Adolescents. Prev Sci.

[3] Haley N, Roy E, Leclerc P, Lambert G, Boivin JF, Cedras L (2002). Risk behaviours and prevalence of Chlamydia trachomatis and Neisseria gonorrhoeae genital infections among Montreal street youth. Int J STD AIDS.

[4] Ringwalt CL, Greene JM, Robertson M, McPheeters M (1998). The prevalence of homelessness among adolescents in the United States. Am J Public Health.

[5] Greene JM, Ringwalt CL (1998). Pregnancy among three national samples of runaway and homeless youth. J Adolesc Health.

[6] Anderson JE, Freese TE, Pennbridge JN (1994). Sexual risk behavior and condom use among street youth in Hollywood. Fam Plann Perspect.

[7] UNAIDS (2009). Condoms and HIV prevention: position statement by UNAIDS, UNFPA and WHO. In.

[8] Crosby R, Warner L (2008). Pending research issues in male condom use promotion. Sex Health.

[9] Seal DW, Ehrhardt AA (2004). HIV-prevention-related sexual health promotion for heterosexual men in the United States: Pitfalls and recommendations. Arch Sex Behav.

[10] De Rosa CJ, Montgomery SB, Hyde J, Iverson E, Kipke MD (2001). HIV risk behavior and HIV testing: A comparison of rates and associated factors among homeless and runaway adolescents in two cities. AIDS Educ Prev.

[11] Tevendale HD, Lightfoot M, Slocum SL (2009). Individual and Environmental Protective Factors for Risky Sexual Behavior among Homeless Youth: An Exploration of Gender Differences. AIDS Behav.

[12] Tucker JS, Ryan G, Golinelli D, Ewing B, Wenzel S, Kennedy DP (2011). Substance Use and Other Risk Factors for Unprotected Sex: Results from an Event-Based Study of Homeless Youth. AIDS Behav.

[13] Halcón LL, Lifson AR (2004). Prevalence and predictors of sexual risks among homeless youth. Journal of Youth and Adolescence.

[14] Bronfenbrenner U (1977). Toward an experimental ecology of human development. Am Psychol.

[15] Sallis JF, Owen N, Fisher EB: Ecological Models of Health Behavior. In: *Heath Behavior and Health Education: Theory, Research and Practice.* 4th edn. Edited by Glanz K, Rimer BK, Viswanath K. San Francisco, CA: Josey-Bass; 2008: 465–482.

[16] Karney BR, Hops H, Redding CA, Reis HT, Rothman AJ, Simpson JA (2010). A Framework for Incorporating Dyads in Models of HIV-Prevention. AIDS Behav.

[17] Naranbhai V, Karim QA, Meyer-Weitz A: Interventions to modify sexual risk behaviours for preventing HIV in homeless youth. Cochrane Database Syst Rev 2011(1) 1-46. 10.1002/14651858.CD007501.pub2 PMC3624078 21249691

[18] Slesnick N, Prestopnik JL (2005). Ecologically based family therapy outcome with substance abusing runaway adolescents. J Adolesc.

[19] Slesnick N, Kang MJ (2008). The impact of an integrated treatment on HIV risk behavior among homeless youth: a randomized controlled trial. J Behav Med.

[20] Burton J, Darbes LA, Operario D (2010). Couples-focused behavioral interventions for prevention of HIV: systematic review of the state of evidence. AIDS Behav.

[21] El-Bassel N, Witte SS, Gilbert L, Wu E, Chang M, Hill J (2003). The efficacy of a relationship-based HIV/STD prevention program for heterosexual couples. Am J Public Health.

[22] Harman JJ, Amico KR (2009). The Relationship-Oriented Information-Motivation-Behavioral Skills Model: A Multilevel Structural Equation Model among Dyads. AIDS Behav.

[23] Misovich SJ, Fisher JD, Fisher WA (1997). Close relationships and elevated HIV risk behavior: evidence and possible underlying psychological processes. Rev Gen Psychol.

[24] Rayburn R, Corzine J (2010). Your Shelter or Mine? Romantic Relationships Among the Homeless. Deviant Behavior.

[25] Kennedy DP, Tucker JS, Green HD, Golinelli D, Ewing BA (2012). Unprotected sex of homeless youth: Results from a multilevel analysis of individual, social network, and relationship factors. AIDS Behav.

[26] Kennedy DP, Wenzel SL, Tucker JS, Green HD, Golinelli D, Ryan GW (2010). Unprotected Sex of Homeless Women Living in Los Angeles County: An Investigation of the Multiple Levels of Risk. AIDS Behav.

[27] Kennedy DP, Wenzel SL, Brown RW, Tucker JS, Golinelli D (2013). Unprotected sex among heterosexually active homeless men: results from a multi-level dyadic analysis. AIDS Behav.

[28] Ryan GW, Stern SA, Hilton L, Tucker JS, Kennedy DP, Golinelli D (2009). When, Where, Why and with Whom Homeless Women Engage in Risky Sexual Behaviors: A Framework for Understanding Complex and Varied Decision-Making Processes. Sex Roles.

[29] Brown RA, Kennedy DP, Tucker JS, Wenzel S, Golinelli D, Wertheimer S (2012). Sex and Relationships on the Street: How Homeless Men Judge Partner Risk on Skid Row. AIDS Behav.

[30] Rana Y, Brown RA, Kennedy DP, Ryan GW, Stern S, Tucker JS: Understanding condom use decision making among homeless youth using event-level data. J Sex Res 2014:1–11.doi:10.1080/00224499.2014.961185 10.1080/00224499.2014.961185 PMC4689140 25396781

[31] Pulerwitz J, Gortmaker SL, DeJong W (2000). Measuring sexual relationship power in HIV/STD research. Sex Roles.

[32] Brown RA, Kennedy DP, Tucker JS, Golinelli D, Wenzel SL (2013). Monogamy on the Street: A Mixed Methods Study of Homeless Men. Journal of Mixed Methods Research.

[33] Tucker JS, Sussell J, Golinelli D, Zhou A, Kennedy DP, Wenzel SL (2012). Understanding pregnancy-related attitudes and behaviors: a mixed-methods study of homeless youth. Perspect Sex Reprod Health.

[34] Bowen AM, Williams M, McCoy HV, McCoy CB (2001). Crack smokers' intention to use condoms with loved partners: Intervention development using the theory of reasoned action, condom beliefs, and processes of change. AIDS Care.

[35] Orr DP, Langefeld CD, Katz BP, Caine VA (1996). Behavioral intervention to increase condom use among high-risk female adolescents. The Journal of Pediatrics.

[36] Power R, French R, Connelly J, George S, Hawes D, Hinton T (1999). Health, health promotion, and homelessness. BMJ.

[37] Toward Understanding Homelessness: The 2007 National Symposium on Homelessness Research [http://aspe.hhs.gov/hsp/homelessness/symposium07/toro/]

[38] Kennedy DP, Brown RA, Golinelli D, Wenzel SL, Tucker JS, Wertheimer S (2012). Masculinity and HIV Risk among Homeless Men in Los Angeles. Psychol Men Masculinity.

[39] Guest G, MacQueen KM (2007). Handbook for Team Based Qualitative Research.

[40] Guest G, MacQueen KM, Namey EE (2011). Applied Thematic Analysis.

[41] Landis JR, Koch GG (1977). The measurement of observer agreement for categorical data. Biometrics.

[42] Afifi WA (1999). Harming the Ones We Love: Relational Attachment and Perceived Consequences as Predictors of Safe-Sex Behavior. The Journal of Sex Research.

[43] Feeney JA, Kelly L, Gallois C, Peterson C, Terry DJ (1999). Attachment style, assertive communication, and safer-sex behavior. J Appl Soc Psychol.

[44] Impett EA, Peplau LA (2002). Why some women consent to unwanted sex with a dating partner: Insights from attachment theory. Psychol Women Q.

[45] Cabral RJ, Pulley L, Artz L, Brill I, Macaluso M (1998). Women at risk of HIV/STD: the importance of male partners as barriers to condom use. AIDS Behav.

[46] Jones R, Oliver M (2007). Young urban women's patterns of unprotected sex with men engaging in HIV risk behaviors. AIDS Behav.

[47] Tucker JS, Elliott MN, Wenzel SL, Hambarsoomian K (2007). Relationship commitment and its implications for unprotected sex among impoverished women living in shelters and low-income housing in Los Angeles county. Health Psychol.

[48] Devries KM, Free C (2010). 'I told him not to use condoms': masculinities, femininities and sexual health of Aboriginal Canadian young people. Sociol Health Illn.

[49] Noar SM, Carlyle K, Cole C (2006). Why Communication Is Crucial: Meta-Analysis of the Relationship Between Safer Sexual Communication and Condom Use. J Health Commun.

[50] Martino S, Tucker J, Ryan G, Wenzel S, Golinelli D, Munjas B (2011). Increased Substance Use and Risky Sexual Behavior Among Migratory Homeless Youth: Exploring the Role of Social Network Composition. Journal of Youth and Adolescence.

[51] Miller WR, Rose GS (2009). Toward a Theory of Motivational Interviewing. Am Psychol.

[52] Green HD, Tucker JS, Wenzel SL, Golinelli D, Kennedy DP, Ryan GW, et al. Association of childhood abuse with homeless women's social networks. Child Abuse & Neglect 2012, 36(1):21–31. 10.1016/j.chiabu.2011.07.005 PMC3659414 22265902

